# Single-Nucleotide Polymorphisms of TAS2R46 Affect the Receptor Downstream Calcium Regulation in Histamine-Challenged Cells

**DOI:** 10.3390/cells13141204

**Published:** 2024-07-16

**Authors:** Giulia Lecchi, Chiara Mocchetti, Davide Tunesi, Arianna Berto, Hari Baskar Balasubramanian, Sima Biswas, Angshuman Bagchi, Federica Pollastro, Luigia Grazia Fresu, Maria Talmon

**Affiliations:** 1 Department of Health Sciences, School of Medicine, University of Piemonte Orientale, Via Solaroli, 17, 28100 Novara, Italy; 2 Department of Biochemistry and Biophysics, University of Kalyani, Kalyani 741235, West Bengal, India; 3 Department of Pharmaceutical Sciences, University of Piemonte Orientale, Largo Donegani 2, 28100 Novara, Italy

**Keywords:** bitter taste receptor, TAS2R, polymorphism, SNPs, calcium signalling

## Abstract

Bitter taste receptors (TAS2Rs) expressed in extraoral tissues represent a whole-body sensory system, whose role and mechanisms could be of interest for the identification of new therapeutic targets. It is known that TAS2R46s in pre-contracted airway smooth muscle cells increase mitochondrial calcium uptake, leading to bronchodilation, and that several SNPs have been identified in its gene sequence. There are very few reports on the structure–function analysis of TAS2Rs. Thus, we delved into the subject by using mutagenesis and in silico studies. We generated a cellular model that expresses native TAS2R46 to evaluate the influence of the four most common SNPs on calcium fluxes following the activation of the receptor by its specific ligand absinthin. Then, docking studies were conducted to correlate the calcium flux results to the structural mutation. The analysed SNPs differently modulate the TAS2R46 signal cascade according to the altered protein domain. In particular, the SNP in the sixth transmembrane domain of the receptors did not modulate calcium homeostasis, while the SNPs in the sequence coding for the fourth transmembrane domain completely abolished the mitochondrial calcium uptake. In conclusion, these results indicate the fourth transmembrane domain of TAS2R46 is critical for the intrinsic receptor activity.

## 1. Introduction

Bitter taste receptors (TAS2Rs) are a family of G protein-coupled receptors which were first described in the oral cavity, exhibiting the ability to sense the bitter taste [1,2,3]. Afterwards, TAS2Rs were found to be distributed throughout the entire body, thus creating a whole-body sensory system [4]. However, the mechanisms behind their functionalities are not yet fully elucidated. Several studies have focused on TAS2Rs expressed in the respiratory systems as a putative alternative target for the treatment of asthma due to their well-established anti-inflammatory and bronchodilating activities [5,6,7]. Nevertheless, a stumbling block in the study of TAS2Rs is to precisely define their mechanism of action. It is established that the signalling cascade downstream of the bitter taste receptors can be divided into two steps. In all cell types the activation of TAS2Rs leads, first of all, to IP_3_-dependent calcium release from the endoplasmic reticulum (ER), but then the fate of this cytosolic calcium differs depending on the bitter taste receptor subtype, the cellular localisation, the ligand, the characteristics of the cells constituting the contracted airway smooth muscle (ASM), and the bronchoconstrictor stimulus [5,6,8,9].

In recent years, we have focused on the identification of a specific inhibitor for bronchoconstriction, and can identify a highly specific agonist, absinthin, which is a sesquiterpene lactone molecule [10]. TAS2R46s are expressed at a lower level in comparison to the other receptor subtypes from which they stand out due to their high sensitivity toward the bitter ligands [5], and this makes them relevant from a translational and pharmacological point of view. Indeed, absinthin is effective at micromolar concentrations, and the absinthin–TAS2R46 binding leads to anti-inflammatory action in bronchial epithelial cells [11] and to a modulation of the cytosolic calcium increased in ASM cells after histamine challenge [12]. In particular, absinthin acts through a striking calcium transient regulation, significantly reducing the cytosolic calcium rise induced by histamine while not reducing the ion flux through the plasma membrane [13] or decreasing Ca^2+^ efflux from the endoplasmic reticulum [13], but rather by increasing mitochondrial Ca^2+^ uptake [12].

From a structural point of view, TAS2Rs are characterised by seven transmembrane domains connected by three intracellular and three extracellular loops that are similar to class A GPCRs [14]. The binding with bitter ligands in the extracellular space and the intracellular signal transmission are critically influenced by the receptor core composed by the transmembrane domains [14,15]. Structure–function analysis identified the seventh transmembrane domain of TAS2R46 as responsible for the agonist selectivity [16]. For example, absinthin specifically binds to the receptor binding pocket through the allylic and homoallylic units that characterise its dimeric guaianolide structure [10], while, for the binding between TAS2R46s and strychnine, the involvement of both an orthosteric and an additional vestibular site have been demonstrated [17,18].

**Figure 1:**
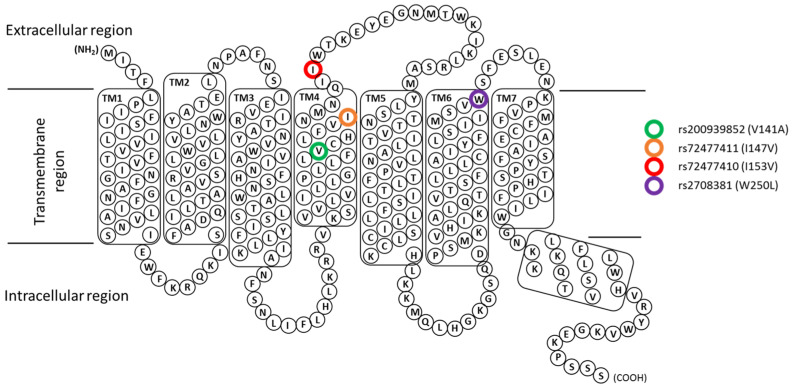
Schematic representation of the amino acid sequence of TAS2R46 with the SNPs considered in this study being highlighted. Snake plot representation of amino acid residues. TAS2R46 consists of a short N terminus (NH2), seven transmembrane domains (TM1-7), three extracellular loops, three intracellular loops, and a short C terminus (COOH). SNPs considered in the present study are marked by coloured circles: rs200939852 (V141A) in green; rs72477411 (I147L) in orange; rs72477410 (I153V) in red; rs2708381 (W250L) in purple.

Since intracellular, extracellular, and transmembrane domains are pivotal in ligand binding and signal transmission, mutations in any amino acid of the total sequence of the receptor could affect both processes. TAS2R gene sequences are strongly characterised by the presence of polymorphisms as a result of a dynamic evolutionary adaptation to specific environments [4,19], and chimeric studies and genotypic–phenotypic association analysis showed that some of these polymorphisms alter bitter perception and the strength of the ligand binding [20]. Additionally, it has been identified that the SNPs in TAS2R38 sequences are responsible for the alterations of human taste perception, behavioural habits, and the incidence of some diseases [21,22] such as upper respiratory infection [23,24,25]. However, since few studies have been performed on the SNPs of the TAS2R46 gene [19,26,27], to deepen the knowledge of its structure and mechanism of action, we have selected four already-known SNPs in transmembrane domains (TM) four and six of the TAS2R46 sequence (Figure 1: TM6: rs2708381 (W250L); TM6: rs72477410 (I153V), rs72477411 (I147V), and rs200936852 (V141A); allele frequency > 0.1), and we have investigated their influence on absinthin binding and the consequent downstream receptor activity using both in silico and in vitro assays, respectively.

## 2. Materials and Methods

### 2.1. Cell Culture

HeLa cells (ATCC; CCL-2) were maintained in Dulbecco’s Modified Eagle Medium (DMEM, Sigma-Aldrich, Milan, Italy) supplemented with 10% heat-inactivated foetal bovine serum (FBS), 1% L-glutamine, and 1% antibiotic–antimycotic (all Thermo Fisher, Monza, Italy) at 37 °C under a 5% CO_2_ humidified atmosphere.

### 2.2. In Vitro Mutagenesis and Generation of TAS2R46-Expressing HeLa Cells

The naïve sequence of the human TAS2R46 gene inserted in a pcDNA3.1-C-(k)DYK was purchased from GeneScript (Clone ID: OHu30358). The plasmid was already edited by in vitro site-directed mutagenesis to insert four selected SNPs of interest based on the allele frequency > 0.1: rs2708381, rs72477410, rs72477411, and rs200936852. The mutagenesis was carried out using the QuikChange Lightning Site-Directed Mutagenesis Kit (Agilent, Milano, Italy) following the manufacturer’s instructions. The mutagenic primers (listed in Table 1) were designed using the web-based QuikChange Primer Design Program (www.agilent.com/genomics/qcpd; accessed on 22 June 2021). The amplification product was checked by Sanger LIGHTrun sequencing from Microsynth AG (Balgach, Switzerland). To generate the cellular models expressing the mutated receptors, 1.2 × 10^6^ HeLa cells were plated in a 10 cm diameter Petri dish and transiently transfected using Lipofectamine 2000 (Invitrogen, Waltham, MA, USA), using 24 µg of each plasmid and 10 µL of Lipofectamine reagent for each dish.

### 2.3. Calcium Imaging

HeLa cells were plated on a pre-coated glass coverslip with 0.1% polilysine. Cytosolic Ca^2+^ fluctuations were evaluated as previously described [12]. Briefly, cells were loaded with 5 µM Fura-2 AM in the presence of 0.02% Pluronic-127 and 10 µM sulfinpyrazone in Krebs–Ringer buffer (KRB) containing 2 mM CaCl2 (30 min, RT); then, they were incubated (20 min, RT) with KRB to allow for the de-esterification of the probe. For the measurements of the mitochondrial Ca^2+^, the cells were loaded with Rhod-2AM in the presence of 0.2% Pluronic-127 in KRB containing 2 mM CaCl_2_ (30 min, RT), and then they were incubated with KRB for 1 h to allow for the de-esterification. The basal calcium was monitored for about 50 s, and then the cells were challenged with the following stimuli, alone or combined: acetylcholine (100 µM; Sigma-Aldrich), absinthin (1, 10, and 100 µM; kindly provided by Dr. Pollastro), forskolin (10 µM; Sigma-Aldrich), and 8-pCPT-2′-O-Me-cAMP (10 µM; Sigma-Aldrich). The coverslips were mounted into an acquisition chamber and placed on the stage of a Leica DMI6000 epifluorescent microscope equipped with an S Fluor ×40/1.3 objective. Fura-2 AM was excited by alternating 340 nm and 380 nm using a Polychrome IV monochromator (Till Photonics, Gräfelfing, Germany), and the probe emission light was filtered through a 520/20 bandpass filter and collected by a cooled CCD camera (Hamamatsu, Japan). Rhod-2AM was excited at 552 nm and the fluorescence emission was recorded at 580 nm. The fluorescence signals were acquired and processed using MetaFluor software 7.8 (Molecular Devices, San Jose, CA, USA). To quantify the differences in the amplitudes of the Ca^2+^ transients, the Fura-2AM and Rhod-2AM fluorescences were expressed relative to the fluorescence intensity at the stimulation time (ΔF/F0).

### 2.4. Model Building and Validations of Wild-Type and Mutant TAS2R46 Models

The Cryo_EM structures of the TAS2R46, represented by the codes 7XP4, 7XP5, and 7XP6 [18], are available in the Protein Data Bank (PDB) (https://www.rcsb.org/; accessed on 25 April 2024).

The details of the structures are as follows:

7XP4: TAS2R46 in apo state;
7XP5: TAS2R46 in ligand-free state;
7XP6: TAS2R46 in active state.

The 7XP6 had some missing residues; therefore, to perform the docking simulations, we filled in these missing residues and generated the final structure of the protein. The amino acid sequence of the protein was retrieved from UniProtKB (www.uniprot.org/; accessed on 25 April 2024), bearing the accession number P59540. The modelled structure of the protein, generated after incorporations of the missing residues, was subjected to energy minimisation in the Discovery Studio 2.5 (D.S 2.5) platform to remove any unwanted steric clashes following the smart minimisation protocol, which is a combination of the Steepest Descent and Conjugate Gradient approaches. The process of minimisation continued until the RMS gradient of the energy derivative reached the value of 0.01 kcal/mole. We then checked the amino acid dispositions in the model by using the SAVES6.0 server (https://saves.mbi.ucla.edu/; accessed on 26 April 2024).

Furthermore, we performed a second round of docking simulations with the 7XP6 without inserting the missing residues. In order to do so, we kept the coordinates of the atoms of the amino acid residues of the TAS2R46 protein only in the 7XP6, and we checked its stereochemical profiles with the help of Ramachandran plots [28]. We then used the Modloop server [29] to correct the loop segments of the protein, and the final structure was built after performing energy minimisation, following the same protocol as previously de-scribed. The mutant structures were built using the ‘Build Mutant’ protocol in D.S 2.5 and refined as per the same protocol mentioned before.

### 2.5. Molecular Docking Simulations

To understand the mode of binding interactions between the TAS2R46 (both the wild-type and mutant receptors) and the ligand absinthin, we used molecular docking simulations with the help of Autodock 4.2 [30]. The structure of the absinthin was retrieved from the PubChem database (https://pubchem.ncbi.nlm.nih.gov/; accessed on 25 April 2024), bearing the PubChem CID: 442138. Additionally, as a control run, we docked TAS2R46 with strychnine, bearing the PubChem CID: 441071. The chosen ligands were subjected to processing to make them suitable for the docking studies with the help of D.S 2.5. We used the flexible docking approach to carry out the molecular docking simulations. It was observed by Sandal et al. [17] and Xu et al. [18] that, along with the orthosteric agonist binding site, another extracellular binding site called the “vestibular” site is also present in the TAS2R46 receptor [17,18]. We then performed the directed docking simulation studies twice, one each for the wild-type and mutants, respectively, creating a box around the binding sites, as mentioned by the authors. The Lamarckian Genetic Algorithm (LGA) was used to perform the docking simulations. Based on the Autodock 4.2 scoring function, the best docking conformations of the protein–ligand complexes with the corresponding lowest binding free energy values were selected. Further interactions of the protein–ligand complexes were analysed by D.S 2.5 and ligplot (https://www.ebi.ac.uk/thornton-srv/software/LigPlus/; accessed on 28 April 2024). The same docking simulation approach was performed for the mutants as well.

### 2.6. Statistical Analysis

For the calcium imaging experiments, statistical analysis was performed by a parametric *t* test/one-way ANOVA or, otherwise, nonparametric alternatives were appropriately used, as reported in the figure captions. Data are presented as mean ± SEM of *n* cells in *n* independent experiments, as detailed in the figure legends. A *p* value < 0.05 was considered statistically significant.

## 3. Results

### 3.1. TAS2R46-HeLa Cells Efficiently Recapitulated the Calcium Movements Downstream of Receptor Activation

**Figure 2:**
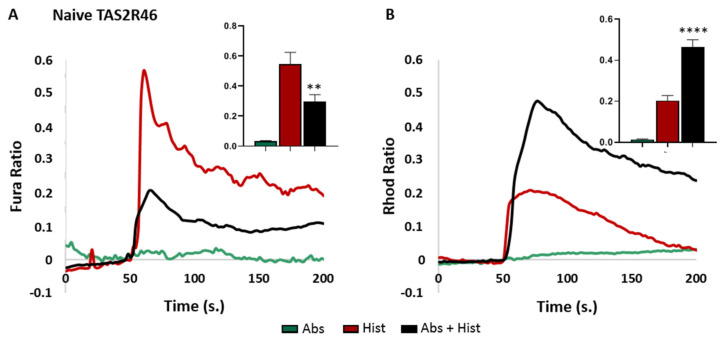
Cytosolic and mitochondrial Ca^2+^ analysis in HeLa cells expressing naive TAS2R46. Fura-2AM- and Rhod-2AM-loaded cells were stimulated with 10 μM of histamine (Hist) and 10 μM of absinthin (Abs) alone or combined. All data are illustrated as representative traces as well as histograms of the maximum peak (inset). (**A**) Cytosolic calcium transient in TAS2R46-expressing HeLa cells. Abs *n* = 33, Hist *n* = 31, and Abs+Hist *n* = 55 cells of 3 independent experiments. (**B**) Mitochondrial calcium analysis in TAS2R46-expressing HeLa cells. Abs *n* = 30 and Hist *n* = 59 cells of 3 independent experiments; Abs+Hist *n* = 134 cells of 4 independent experiments. Statistical analysis: Mann–Whitney U test; ** *p* < 0.01 and **** *p* < 0.0001 vs. Hist.

As previously demonstrated in the ASM cells, the binding between absinthin and TAS2R46 mediates the bronchodilation of histamine-challenged cells, driving the calcium shift from the ER to the mitochondria [12]. To ascertain the functional consequence on the receptor activity of the four mutations, wild-type hTAS2R46 and mutant receptors were expressed in HeLa cells, and mitochondrial and cytosolic calcium variations were quantitated in real time for about 50 s after the exposure of the cells to histamine and/or absinthin. First of all, we confirmed by immunofluorescence the goodness of the transfection (Figure S1); then, with a functional assay, we validated the TAS2R46-expressing HeLa cells as a good model to recap the downstream calcium modulation. Indeed, the non-transfected cells had no detectable calcium response either after treatment with absinthin alone or after co-treatment with histamine (Figure S2), while the wild-type TAS2R46-expressing cells showed a rapid increase in the cytosolic calcium concentration upon histamine challenge, which was promptly reversed by absinthin (Figure 2A), with a parallel significant increase in the mitochondrial calcium uptake (Figure 2B).

### 3.2. Effects of the Polymorphism on Downstream Signalling

As previously described in Figure 1, the amino acid mutations induced by each SNP are as follows: the rs2708381 polymorphism determines the mutation of the Trp250 into a Leu (W250L) or introduces a Stop codon in the sixth transmembrane domain, while the rs72477410, rs72477411, and rs200936852 polymorphisms lead to mis-sense variants in I153V, I147V, and V141A, respectively, in the fourth transmembrane domain. We therefore compared the signal transduction in native-receptor-expressing cells and those expressing the SNPs to understand the impact a mutation may have on the ligand binding or activation of the downstream signalling pathway.

**Figure 3:**
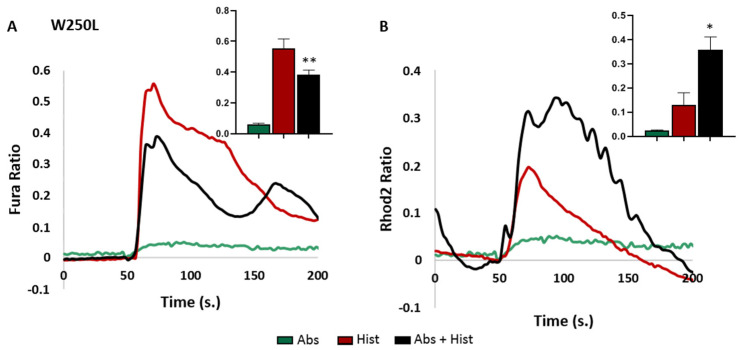
Cytosolic and mitochondrial Ca^2+^ analysis in HeLa cells expressing W250L-TAS2R46. Fura-2AM- and Rhod-2AM-loaded cells were stimulated with 10 μM of histamine (Hist) and 10 μM of absinthin (Abs) alone or combined. All data are illustrated as representative traces as well as histograms of the maximum peak (inset). (**A**) Cytosolic calcium transient in rs2708381 TAS2R46-expressing HeLa cells. Abs *n* = 28, Hist *n* = 51, and Abs+Hist *n* = 73 cells of 3 independent experiments. (**B**) Mitochondrial calcium analysis in rs2708381 TAS2R46-expressing HeLa cells. Abs *n* = 24, Hist *n* = 26, and Abs+Hist *n* = 26 cells of 3 independent experiments. Statistical analysis: Mann–Whitney U test; * *p* < 0.05 and ** *p* < 0.01 vs. Hist.

As shown in Figure 3, the response to the histamine/absinthin co-treatment of the W250L mis-sense variant in TM6 was superimposable to that of the wild-type.

**Figure 4:**
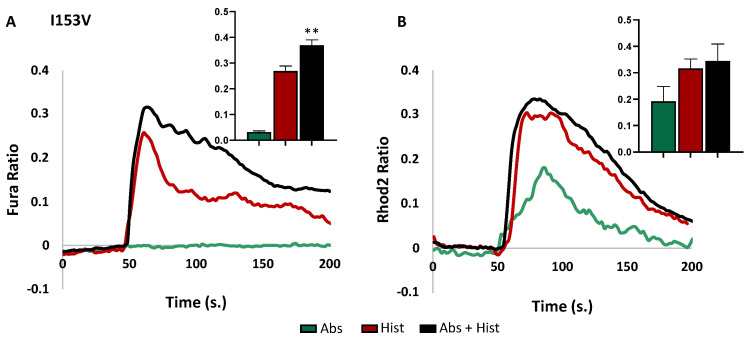

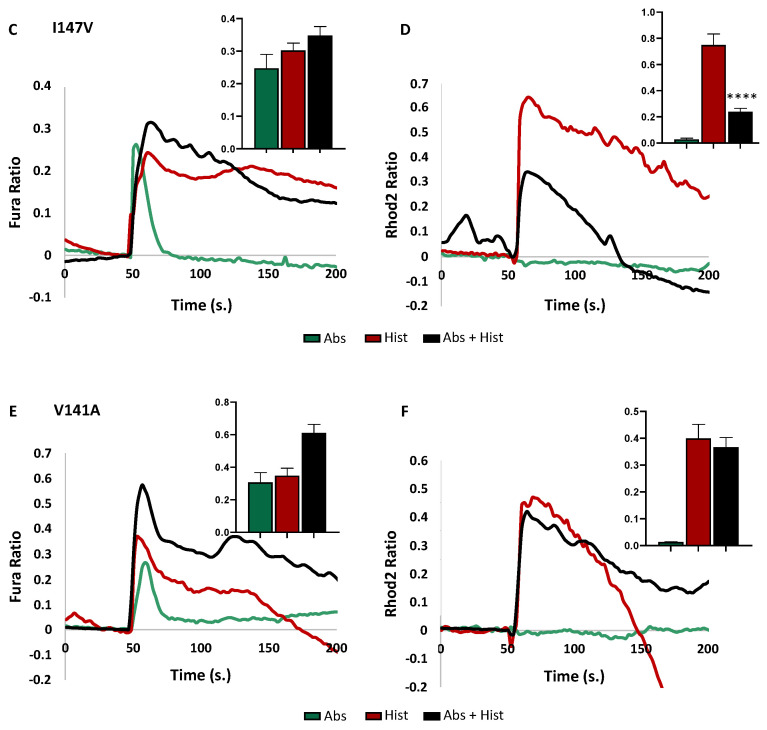
Cytosolic and mitochondrial Ca^2+^ analysis HeLa cells expressing I153V-, I147V-, and V141A-TAS2R46. Fura-2AM- and Rhod-2AM-loaded cells were stimulated with 10 μM of histamine (Hist) and 10 μM of absinthin (Abs) alone or combined. All data are illustrated as representative traces as well as histograms of the maximum peak (inset). (**A**) Cytosolic calcium transient in I153V-HeLa cells. Abs *n* = 47 cells of 3 independent experiments; Hist *n* = 81 and Abs+Hist *n* = 114 cells of 4 independent experiments. (**B**) Mitochondrial calcium analysis in I153V-HeLa cells. Abs *n* = 18, Hist *n* = 41, and Abs+Hist *n* = 29 cells of 3 independent experiments. (**C**) Cytosolic calcium transient in I147V-HeLa cells. Abs *n* = 46, Hist *n* = 38, and Abs+Hist *n* = 57 cells of 3 independent experiments. (**D**) Mitochondrial calcium analysis in I147V-HeLa cells. Abs *n* = 27, Hist *n* = 31, and Abs+Hist *n* = 48 cells of 3 independent experiments. (**E**) Cytosolic calcium transient in V141A-HeLa cells. Abs *n* = 38, Hist *n* = 36, and Abs+Hist *n* = 55 cells of 3 independent experiments. (**F**) Mitochondrial calcium analysis in V141A-HeLa cells. Abs *n* = 40, Hist *n* = 46, Abs+Hist *n* = 39 cells of 3 independent experiments. Statistical analysis: Mann–Whitney U test; ** *p* < 0.01 and **** *p* < 0.0001 vs. Hist.

In contrast, mis-sense variants in I153V, I147V, and V141A in TM4 affected the receptor activity, with different consequences depending on the mutation site (Figure 4). Unexpectedly, we observed that, in I153V-HeLa cells, absinthin alone did not alter the basal cytosolic calcium level, while it did induce an increase in mitochondrial calcium (Figure 4A,B). Conversely, the other variants induced cytosolic calcium peaks (Figure 4C–F). Moreover, several of the SNPs also differentially affected the modulation of histamine calcium release. In fact, in I153V-HeLa cells, co-stimulation with absinthin and histamine significantly increased, rather than decreased, the calcium levels in the cytosol (Figure 4A), while no changes were recorded in the mitochondria compared with the stimulation of histamine alone (Figure 4B). In I147V-HeLa cells, absinthin was not able to revert the histamine-induced Ca^2+^ transient (Figure 4C), but the calcium uptake in the mitochondria was significantly lower in the cells stimulated with absinthin and histamine compared to histamine alone (Figure 4D). Finally, in the V141A-HeLa cells, absinthin synergised with histamine (Figure 4E), with no consequent effect recorded in the mitochondria (Figure 4F).

### 3.3. EPAC Activation Rescued the Absinthin Effect Downstream of Mutated Receptors

**Figure 5:**
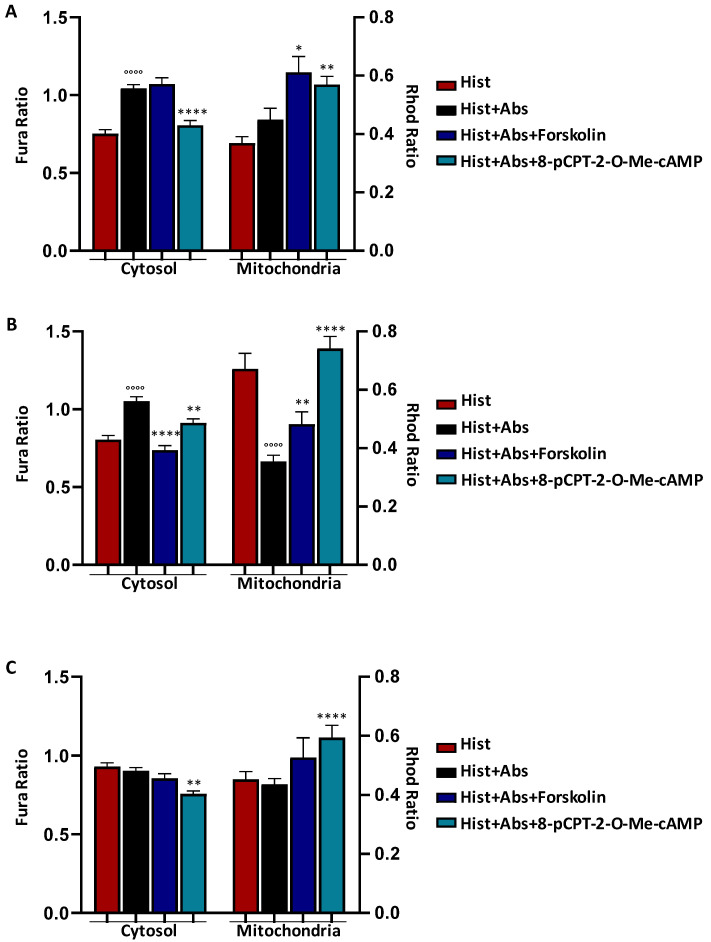
EPAC activation in HeLa cells expressing I153V-, I147V-, and V141A-TAS2R46. Fura-2AM- and Rhod-2AM-loaded cells were stimulated with 10 μM of histamine (Hist), 10 μM of absinthin (Abs), 10 μM of forskolin, and 10 μM of 8-pCPT-2-O-Me-cAMP. All data are illustrated as histograms showing the mean ± SEM of the maximum peak. (**A**) Cytosolic (Hist *n* = 219 cells; Hist+Abs *n* = 250; Hist+Abs+Forsk *n* = 175; Hist+Abs+8-pCPT-2-O-Me-cAMP *n* = 145) and mitochondrial (Hist *n* = 110 cells; Hist+Abs *n* = 109; Hist+Abs+Forsk *n* = 102; Hist+Abs+8-pCPT-2-O-Me-cAMP *n* = 133) calcium transient in I153V-HeLa cells. (**B**) Cytosolic (Hist *n* = 271 cells; Hist+Abs *n* = 219; Hist+Abs+Forsk *n* = 154; Hist+Abs+8-pCPT-2-O-Me-cAMP *n* = 254) and mitochondrial (Hist *n* = 71 cells; Hist+Abs *n* = 123; Hist+Abs+Forsk *n* = 59; Hist+Abs+8-pCPT-2-O-Me-cAMP *n* = 45) calcium transient in I147V-HeLa cells. (**C**) Cytosolic (Hist *n* = 336 cells; Hist+Abs *n* = 328; Hist+Abs+Forsk *n* = 281; Hist+Abs+8-pCPT-2-O-Me-cAMP *n* = 323) and mitochondrial (Hist *n* = 246 cells; Hist+Abs *n* = 204; Hist+Abs+Forsk *n* = 90; Hist+Abs+8-pCPT-2-O-Me-cAMP *n* = 205) calcium transient in V141A-HeLa cells. Statistical analysis: Mann–Whitney U test; °°°° *p* < 0.0001 vs. Hist; * *p* < 0.05, ** *p* < 0.01, and **** *p* < 0.0001 vs. Hist+Abs.

The Exchange Protein Directly Activated by cAMP (EPAC) localises into the mitochondrial inner membrane and may control the mitochondrial calcium uniport (MCU) [31,32]. We have previously demonstrated that absinthin induces calcium shuttling into the mitochondria by activating the EPAC [12]. In naive HeLa cells, absinthin was ineffective against histamine-induced calcium rise, confirming that the effect on calcium modulation is TAS2R46-dependent (Figure S3) and, as previously demonstrated [12,33], the co-stimulation of histamine and forskolin (an adenylate cyclase activator) or the 8-pCPT-2-O-Me-cAMP (a selective EPAC activator) decreased the cytosolic calcium release (Figure S3). Then, in order to evaluate the interference of amino acid mutations in the fourth transmembrane (I153V, I147V, and V141A) domain on the cAMP-mediated signalling triggered by absinthin, we challenged transfected cells with forskolin and 8-pCPT-2-O-Me-cAMP (Figure 5). In line with Figure 4, we observed that absinthin was unable to counteract the histamine-induced cytosolic calcium release in transfected cells with the three variants (Figure 5A–C, right graphs). On the contrary, the addition of 8-pCPT-2-O-Me-cAMP, along with histamine and absinthin, caused a decrease in the cytosolic calcium peak and increased the mitochondrial calcium (Figure 5), while the co-stimulation with forskolin showed an effect only in the I147V-HeLa cells, suggesting that the calcium modulation was mainly mediated by the EPAC. These data therefore corroborate the hypothesis that these mutations interfered with the trigger of the cAMP-EPAC signalling cascade induced by the binding of absinthin to TAS2R46.

### 3.4. Structural Analysis

**Figure 6:**
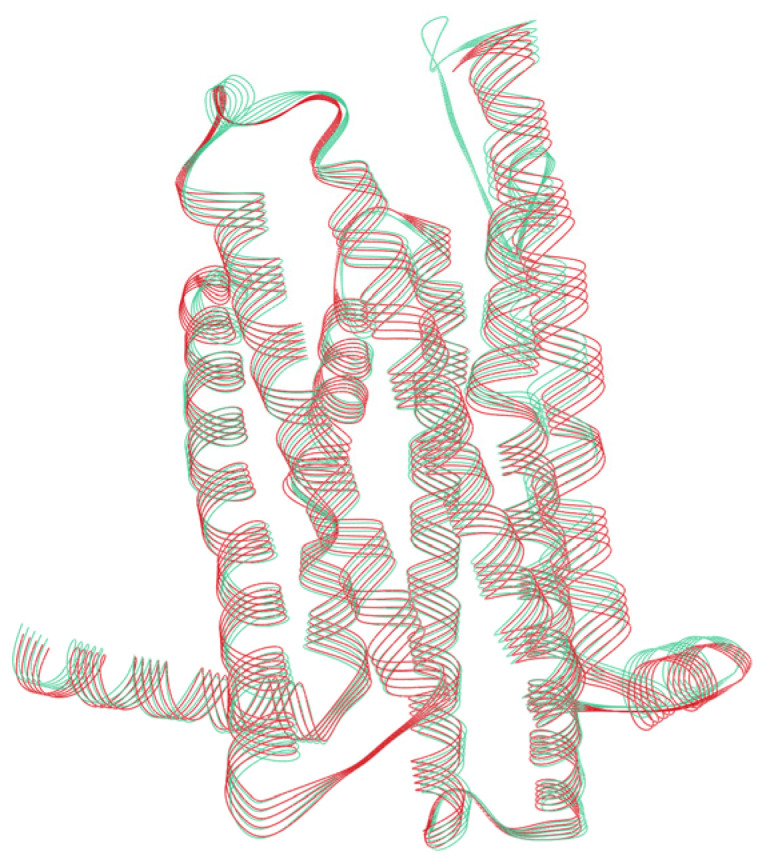
Structural comparison of the TAS2R46 model structure with 7XP6. The rust-red colour denotes the 7XP6 structure (Cryo_EM) and the sap-green colour denotes the modelled structure of TAS2R46.

To understand whether the impairment of the downstream signal transduction was due to an alteration in the binding strength induced by the mutations, we performed two rounds of docking simulations. In the first round, we used the modelled structure of the TAS2R46 obtained after the insertions of the missing amino acid residues in the 7XP6. We made a structural comparison between the model and the 7XP6, and the RMSD of the backbone atoms was found to be 0.781Å (Figure 6). This would indicate that the model and the 7XP6 had similar structural organisations.

**Figure 7:**
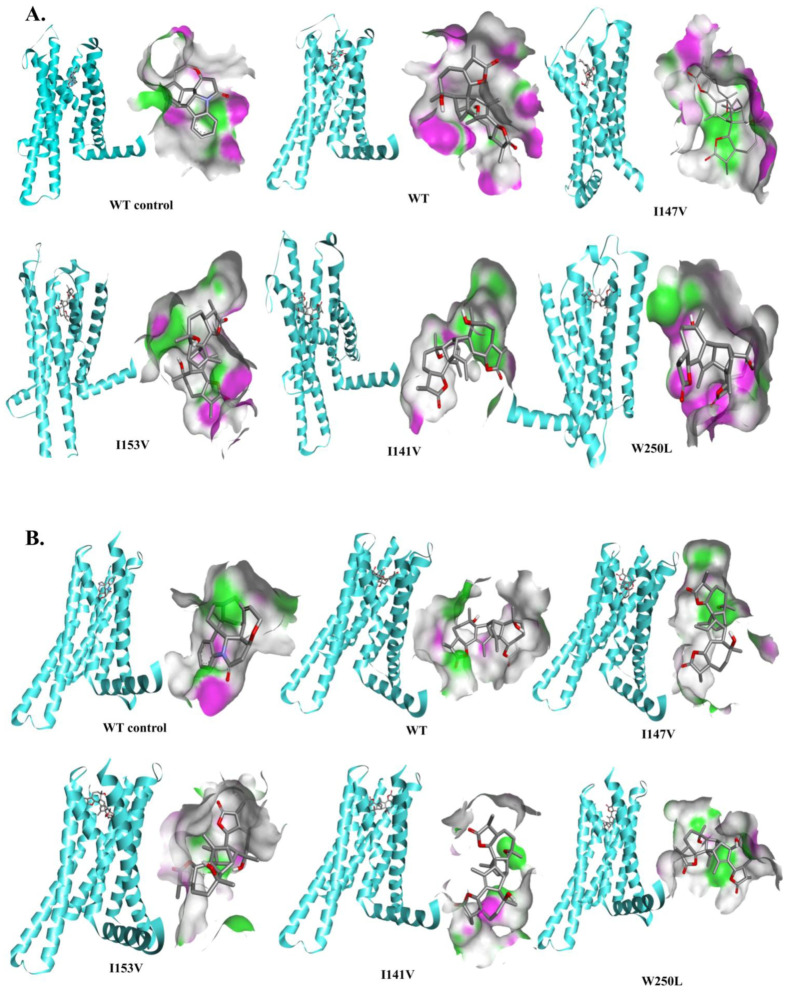
Structural analysis—Orthosteric site. (**A**) Modelled TAS2R46; (**B**) 7XP6: TAS2R46. WT control: structural analysis between the WT receptor and strychnine; WT, I147V, I153V, I141V, and W250L: structural analysis between TAS2R46 and absinthin. The binding interactions between the wild type and the mutant TAS2R46 proteins are presented as follows: the receptor is presented by secondary type while the ligand is shown in ball & stick. The interacting residues are presented in cloud form and depicted side-by-side.

**Figure 8:**
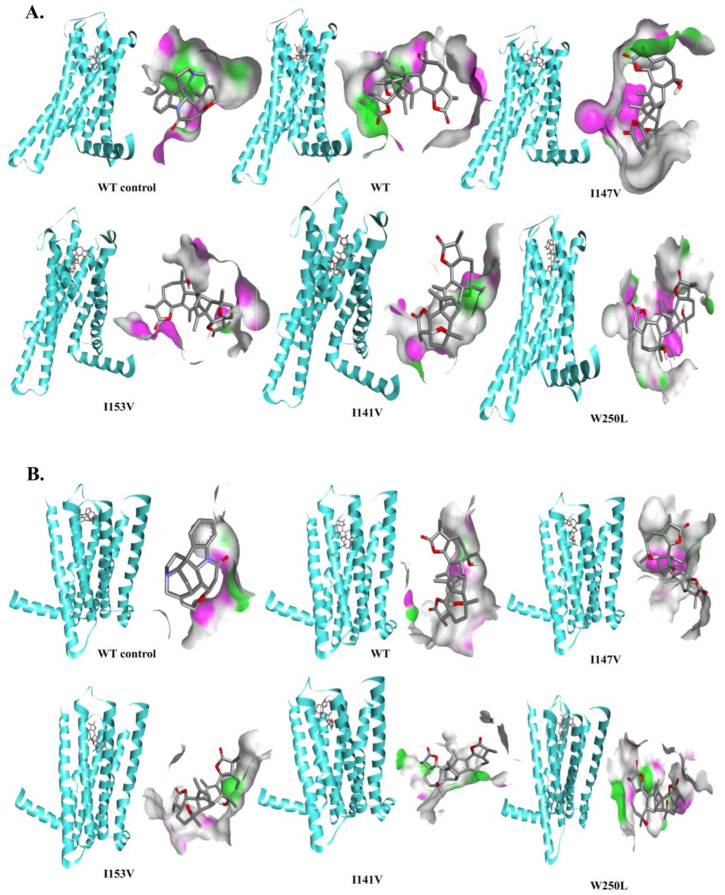
Structural analysis—Vestibular site. (**A**) Modelled TAS2R46; (**B**) 7XP6: TAS2R46. WT control: structural analysis between the WT receptor and strychnine; WT, I147V, I153V, I141V, and W250L: structural analysis between TAS2R46 and absinthin. The binding interactions between the wild type and the mutant TAS2R46 proteins are presented as follows: the receptor is presented by secondary type while the ligand is shown in ball & stick. The interacting residues are presented in cloud form and depicted side-by-side.

We proceeded with a computational analysis based on the flexible docking simulations of absinthin with the wild-type (WT) and mutant TAS2R46 proteins both to the orthosteric (Figure 7) and vestibular sites (Figure 8), and compared the results with the binding interaction profile between strychnine and the WT receptor. We selected the best binding poses (Figure 7 and Figure 8) with the lowest binding free energy values as obtained from Autodock 4.2.

As shown in Table 2, at the orthosteric site, the binding free energy values of absinthin with the wild-type (−10.91 kcal/mol) and the two mutant (W250L and I147V) structures of TAS2R46 (model) are similar. The energy values are slightly higher in the mutants I153V and V141A. Moreover, the binding interaction profiles are also different in the cases of these two mutants.

On the other hand, the binding free energy value of wild-type TAS2R46 (model) with strychnine is −8.68 kcal/mol. Thereafter, we went on to check the binding free energy value obtained after the docking of strychnine directly with the 7XP6, where the process of the insertions of the missing residues were not performed, and it was found to be −9.63 kcal/mol. In these two cases, similar patterns of binding interaction profiles between strychnine and TAS2R46 were also observed.

Interestingly, the patterns of the bindings in all of the complexes were somewhat similar, and the wild-type and mutant receptors shared a similar amino acid distribution in their zones of binding for the ligand absinthin. (Figure 7). However, in some cases their modes of bond formations were different. Similar trends in binding interactions were observed in both types of the aforementioned docking simulations.

Furthermore, we explored the binding interactions of absinthin to the vestibular binding site (Table 3). In this case, we found that the binding free energy values of the wild-type and mutant TAS2R46 proteins (model) were also similar, and that the mutant I147V showed a lower affinity than the wild-type protein. However, the binding affinity of strychnine was higher towards the orthosteric site (Figures S4 and S5). Surprisingly, when we docked strychnine in the vestibular site, the binding affinity was significantly lower than that of absinthin. Furthermore, strychnine interacted with different sets of amino acid residues (Figures S6 and S7). A possible reason behind this is the different chemical structure between the absinthin and strychnine, which therefore might lead the ligands to bind in different ways. Similar results were observed by performing both of the aforementioned docking simulations.

## 4. Discussion

The present report suggests the involvements of the fourth transmembrane domain (TM4) of TAS2R46 as the fundamental unit for the receptor activation by its ligand absinthin. A deeper understanding of the biology of the bitter taste receptor is a great challenge and is currently reliant on in silico and in vitro functional analyses. In fact, the TAS2Rs show a very low sequence and structural similarity with other GPCRs [14,34], which is why the TAS2Rs have been considered to belong to a distinct family [35] or have been grouped with frizzled receptors [36], but most of the studies classify them with the rhodopsin-like receptors (Class A GPCRs) [37,38,39]. TAS2R46, one of the 25 subtypes belonging to the bitter receptor family, has been studied by cryo-electron microscopy [18], computational analysis, and mutagenesis [16,17], which could help in deciphering its structure and biological response to ligands. However, most studies concern the binding between strychnine (a non-selective agonist for TAS2R and an antagonist of the glycine receptor) and TAS2R46, demonstrating that this receptor subtype is characterised by a particular structural rearrangement of TM3, TM4, and TM5 that leads to the formation of a pocket near the bilayer [18]. Furthermore, Sandal and colleagues identified a vestibular site involved in strychnine binding in addition to the classical orthosteric one [17]. Moreover, it is well reported that the domains critical to the receptor binding and activity vary depending on the bitter ligand [40].

In the present work, we investigated the behavioural binding of absinthin, a selective agonist for the bitter taste receptors of TAS2R46 [41], for which no affinity towards other receptors has been reported and whose ability to hamper calcium increase when triggered by a contraction stimulus (e.g., histamine) is well demonstrated [12]. We therefore focused on the binding interactions between TAS2R46 and absinthin by in silico and in vitro approaches, analysing the four most represented SNPs in the European population, to evaluate any changes in the signal transduction resulting from impaired ligand binding due to the mutations. Indeed, since the TAS2Rs share a high sequence homology among themselves, but show different affinities toward several ligands, a single amino acid substitution could determine a significant change in the receptor behaviour. As shown by our results, although the TM6 and TM7 have been previously demonstrated to be crucial for the ligand selectivity [16], the polymorphism affecting the 250th amino acid residue in TM6 did not alter the calcium modulation in histamine-challenged cells, demonstrating that, probably, this mutation could just reduce the affinity towards absinthin without interfering with the receptor activity. The docking study strengthened our observation, since the binding free energy values of wild-type TAS2R46 and the mutants affecting both TM6 and TM4 do not vary significantly, suggesting that the mutations do not induce drastic changes in the physico-chemical characteristics of the concerned amino acid residues involved in binding absinthin.

Interestingly, the in silico analysis demonstrated that absinthin binds with both the orthosteric and vestibular sites of the wild-type TAS2R46 with almost the same binding affinity, but the mutation I153V in the TM4 negatively affected the TAS2R46–absinthin interactions as compared to the wild-type. This might be due to the differences in the patterns of bond formations in both the orthosteric and vestibular sites of TAS246R in the case of the aforementioned mutant. The apparent consequence of this altered binding is an altered intrinsic activity of absinthin manifested by the different modulation of calcium homeostasis. Moreover, in the mutant I147V, no significance changes were observed in both of the binding sites, but the mode of the bindings may have affected the calcium modulation due to an additional interaction with Asn65. Therefore, the results coming from the docking studies would complement the results from the in vitro experiments.

It is well established that, in GPCR class A, the structural core of the receptor composed of the seven transmembrane domains is highly conserved and crucial for both ligand binding and intracellular signal transduction [14,15]. Thus, it is conceivable that, as for other members of GPCR class A [37,40,42], the SNPs in the TM4 domain of TAS2R46 lead to a rearrangement of the receptor core, leading to an altered downstream transmission. Indeed, this may be the case since the stimulation of these cells with a specific EPAC activator is sufficient to rescue the absinthin/TAS2R46 effect on mitochondrial calcium uptake. This is in line with the already published results that highlighted the role of the EPAC in mediating TAS2R46 signalling in several cell types, such as smooth muscles [12], skeletal muscles [33], and the heart [32].

Based on our results, we can speculate that the TM4, even if it is not directly involved in ligand specificity [10], is fundamental for absinthin activity, but more functional studies (e.g., dose–response curves) must be conducted on mutated or chimeric receptors to respond to our hypothesis.

However, our preliminary results are helpful for the understanding of the structure and the biology of TAS2Rs, an almost stand-alone GPCR class, in order to validate these receptors as new potential targets for respiratory disease therapy.

## Figures and Tables

**Table 1:** Primers for mutagenesis reactions.

Primer Name	Sequence	Tm (°C)
rs2708381 fw	5′-TTTTCCAGACTCTCAAAACTCWAAACTGACATGATTATGGACAG-3′	78.8
rs2708381 rev	5′-CTGTCCATAATCATGTCAGTTTWGAGTTTTGAGAGTCTGGAAAA-3′	78.8
rs72477410 fw	5′-TTCATATTCTTTTGTCCATACAATCTGATTCATGTTTATCACAAAAAGATGACAAACC-3′	79.5
rs72477410 rev	5′-GGTTTGTCATCTTTTTGTGATAAACATGAATCAGATTGTATGGACAAAAGAATATGAA-3′	79.5
rs72477411 fw	5′-TTGTCCATATAATCTGATTCATGTTTACCACAAAAAGATGACAAACCAAAAATAG-3′	78.6
rs72477411 rev	5′-CTATTTTTGGTTTGTCATCTTTTTGTGGTAAACATGAATCAGATTATATGGACAA-3′	78.6
rs200936852 fw	5′-TGTTTATCACAAAAAGATGACAAGCCAAAAATAGCAAAGGCCCCAA-3′	78.9
rs200936852 rev	5′-TTGGGGCCTTTGCTATTTTTGGCTTGTCATCTTTTTGTGATAAACA-3′	78.9

**Table 2:** Autodock binding free energy values of wild-type and mutant TAS2R46 proteins (model and 7XP6) based on their bindings onto the orthosteric site. Model: Modelled structure of TAS2R46 obtained after insertions of the missing residues in 7XP6.

Orthosteric Site		
Name of the Protein–Ligand Complex	Binding Free Energy (kcal/mol)	Inhibition Constant (µM)
Model_WT_strychnine	−8.68	0.43
Model_WT_absinthin	−10.91	0.01
Model_I147V_absinthin	−11.77	0.002
Model_I153V_absinthin	−12.59	0.0005
Model_V141A_absinthin	−12.54	0.0006
Model_W250L_absinthin	−11.01	0.008
7XP6_WT_strychnine	−9.63	0.087
7XP6_WT_absinthin	−12.10	0.001
7XP6_I147V_absinthin	−12.18	0.001
7XP6_I153V_absinthin	−11.40	0.004
7XP6_V141A_absinthin	−12.01	0.001
7XP6_W250L_absinthin	−10.77	0.012

**Table 3:** Autodock binding free energy values of wild-type and mutant TAS2R46 proteins (model and 7XP6) based on their bindings onto the vestibular site. Model: Modelled structure of TAS2R46 obtained after insertions of the missing residues in 7XP6.

Vestibular Site		
Name of the Protein–Ligand Complex	Binding Free Energy (kcal/mol)	Inhibition Constant (µM)
7XP6_WT_strychnine	−5.21	152.25
7XP6_WT_absinthin	−8.16	1.04
7XP6_I147V_absinthin	−7.58	2.80
7XP6_I153V_absinthin	−9.82	0.63
7XP6_V141A_absinthin	−8.10	1.15
7XP6_W250L_absinthin	−8.35	0.76
Model_WT_strychnine	−8.01	1.34
Model_WT_absinthin	−9.60	0.09
Model_I147V_absinthin	−7.26	4.77
Model_I153V_absinthin	−9.59	0.092
Model_V141A_absinthin	−8.12	1.12
Model_W250L_absinthin	−7.83	1.83

## Data Availability

The datasets analysed during the current study are not present in a database but are available from the corresponding author upon request.

## Supplementary Materials

The following supporting information can be downloaded at: https://www.mdpi.com/article/10.3390/cells13141204/s1, Supplemental Material S1: Immunofluorescence; Figure S1: Immunofluorescence staining on transfected HeLa cells; Figure S2: Cytosolic and mitochondrial Ca^2+^ analysis in non-transfected HeLa cells; Figure S3: Cytosolic and mitochondrial Ca^2+^ analysis of non-transfected HeLa cells; Figure S4: Structural analysis; Figure S5: Interaction of modelled TAS2R46 with strychnine and absinthin in orthosteric site; Figure S6: Interaction of 7XP6 TAS2R46 with strychnine and absinthin in vestibular site; Figure S7: Interaction of modelled TAS2R46 with strychnine and absinthin in vestibular site.
