# Supplementary material for: Single-Nucleotide Polymorphisms of TAS2R46 Affect the Receptor Downstream Calcium Regulation in Histamine-Challenged Cells
Source: Cells. 2024 Jul 16;13(14):1204. doi: 10.3390/cells13141204 (PMC11275237; doi:10.3390/cells13141204)
Supplement: Supplementary file 1 [file cells-13-01204-s001.zip › cells-3024967 supplementary.pdf]

SNPs of TAS2R46 affect the receptor downstream calcium regulation in histamine challenged cells

Giulia Lecchi[1], Chiara Mocchetti[1], Davide Tunesi[1], Arianna Berto[1], Hari Baskar Balasubramanian[1], Sima Biswas[2], Angshuman Bagchi[2], Federica Pollastro[3], Luigia Grazia Fresu[1*], Maria Talmon[3*]

[1] Department of Health Sciences, School of Medicine, University of Piemonte Orientale, Via Solaroli, 17 - 28100 Novara, Italy.
[2] Department of Biochemistry and Biophysics, University of Kalyani, Kalyani -741235, Nadia, West Bengal, India.
[3] Department of Pharmaceutical Sciences, University of Piemonte Orientale, Largo Donegani 2 – 28100 Novara, Italy.
* Correspondence: M.T.: maria.talmon@med.uniupo.it; L.G.F.: luigia.fresu@med.uniupo.it.

Supplemental material

Immunofluorescence

To evaluate native and mutated TAS2R46 expression, immunofluorescence for the receptor and plasmid tag has been performed. 2×10^5 cells were plated on 12 mm diameter glass dish. The cells were fixed (PAF 4%, 10 min, 4°C) and then incubated with the blocking buffer (3% BSA, 0.1% Triton X-100 in PBS, 1 h, RT). The cells were incubated with primary antibody for 2h at RT and then 45 min RT in dark with secondary antibodies. The primary antibodies used were the polyclonal rabbit anti-human TAS2R46 (Thermo Fisher) and the mouse monoclonal anti-DYKDDDDK Tag antibody (GenScript). The secondary antibodies were goat anti-rabbit AlexaFluor 488 and goat anti-mouse AlexaFluor 546 (Thermo Fisher), respectively. For nuclei detection DAPI was added to the secondary antibody's solution. Images were acquired and quantified using Leica LAS-X software.

Supplementary figure 1

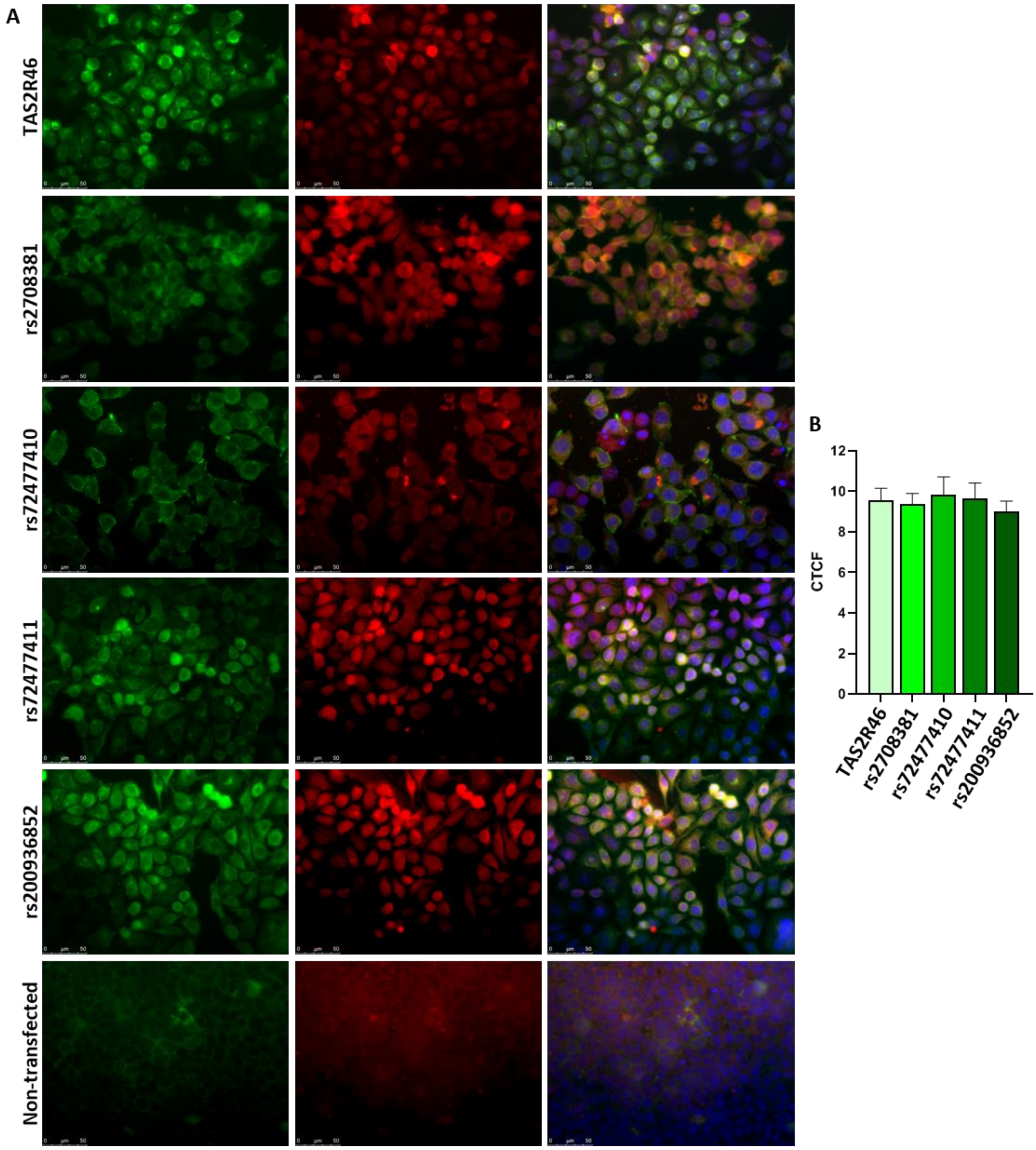

Figure S1. Immunoflorescence staining on transfected HeLa cells. HeLa cells were transfected with TAS2R46 native and mutated constructs and stained by immunofluorescence. A) Rapresentative images. TAS2R46 in green, DYKDDDDK tag in red and nuclei stained with DAPI (blue). Magnifcation 200x. B) quantification of TAS2R46 membrane fluorescence intensity. Data are expressed ad mean±SEM of integrated density (CTCF) of at least 30 cells.

Supplementary figure 2

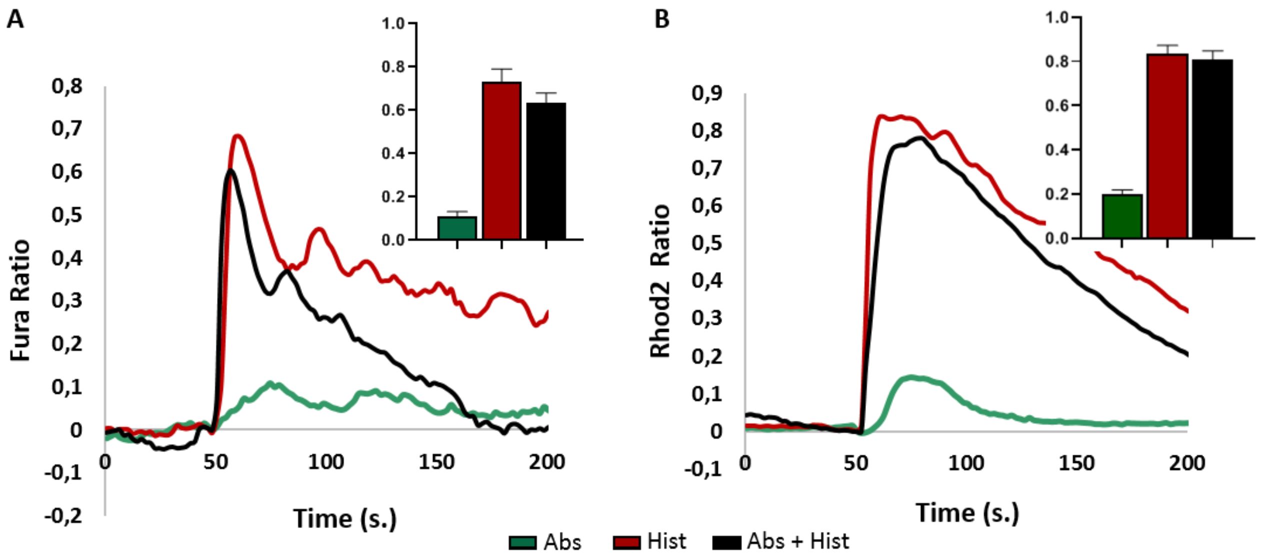

Figure S2. Cytosolic and mitochondrial Ca^{2+} analysis in non-transfected HeLa cells. Fura-2AM- and Rhod-2AM-loaded cells were stimulated with histamine (Hist) 10 µm and absinthin (Abs) 10 µm alone or combined. All data are illustrated as representative traces as well as histograms of the maximum peak (inset). A) Cytosolic calcium transient in HeLa cells. Abs n=59 cells of 3 independent experiments; Hist n=110 and Abs+Hist n=126 cells of 5 independent experiments. B) Mitochondrial calcium analysis in TAS2R46-expressing HeLa cells. Abs n=154; Hist n=211; Abs+Hist n= 166 cells of 6 independent experiments.

Supplementary figure 3

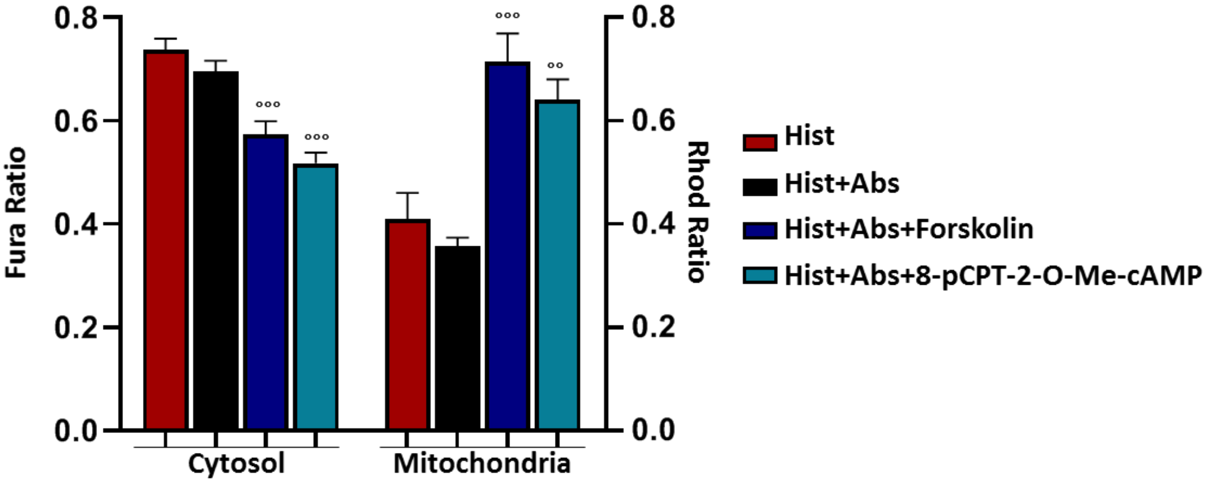

Figure S3. Cytosolic and mitochondrial Ca^{2+} analysis non-transfected HeLa cells. Fura-2AM- and Rhod-2AM-loaded cells were stimulated with histamine (Hist) 10 µm, absinthin (Abs) 10 µm, forskolin 10 µm and 8-pCPT-2-O-Me-cAMP 10 10 µm. All data are illustrated as histograms showing the mean ± SEM of the maximum peak. Cytosolic (Hist n=193 cells; Hist+Abs n=223; Hist+Abs+Forsk n=150; Hist+Abs+8-pCPT-2-O-Me-cAMP n=154) and mitochondrial (Hist n=83 cells; Hist+Abs n=146; Hist+Abs+Forsk n=80; Hist+Abs+8-pCPT-2-O-Me-cAMP n=148) calcium transient in non-transfected HeLa cells. Statistical analysis: Mann–Whitney U test; **p<0.01 and ***P<0.001 vs Hist.

Supplementary figure 4

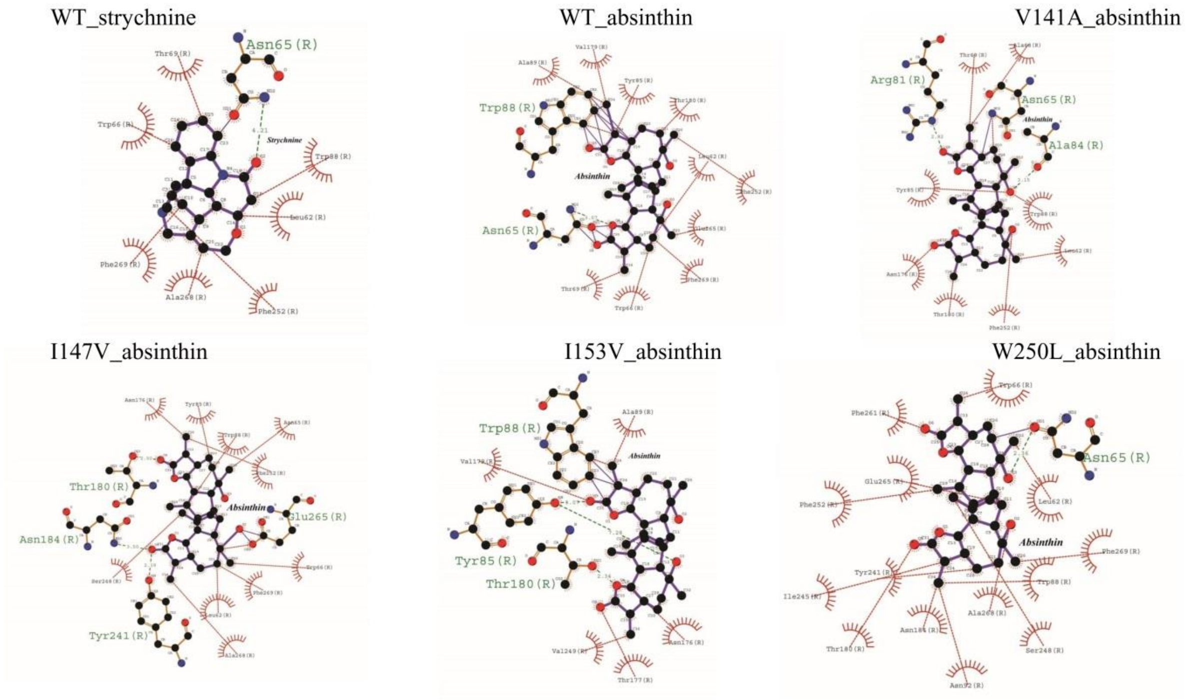

Figure S4. Interaction of 7XP6 TAS2R46 with strychnine and absinthin in orthosteric site. This figure depicts the modes of interactions of the cryo EM structure of TAS2R46 (wild type and mutants) with strychnine and absinthin in orthosteric site. The ligplot analyses show the hydrogen bonding (green dashed lines) and hydrophobic (red dot) interaction profiles of the wild type and mutant TAS2R46 proteins with the ligands.

Supplementary figure 5

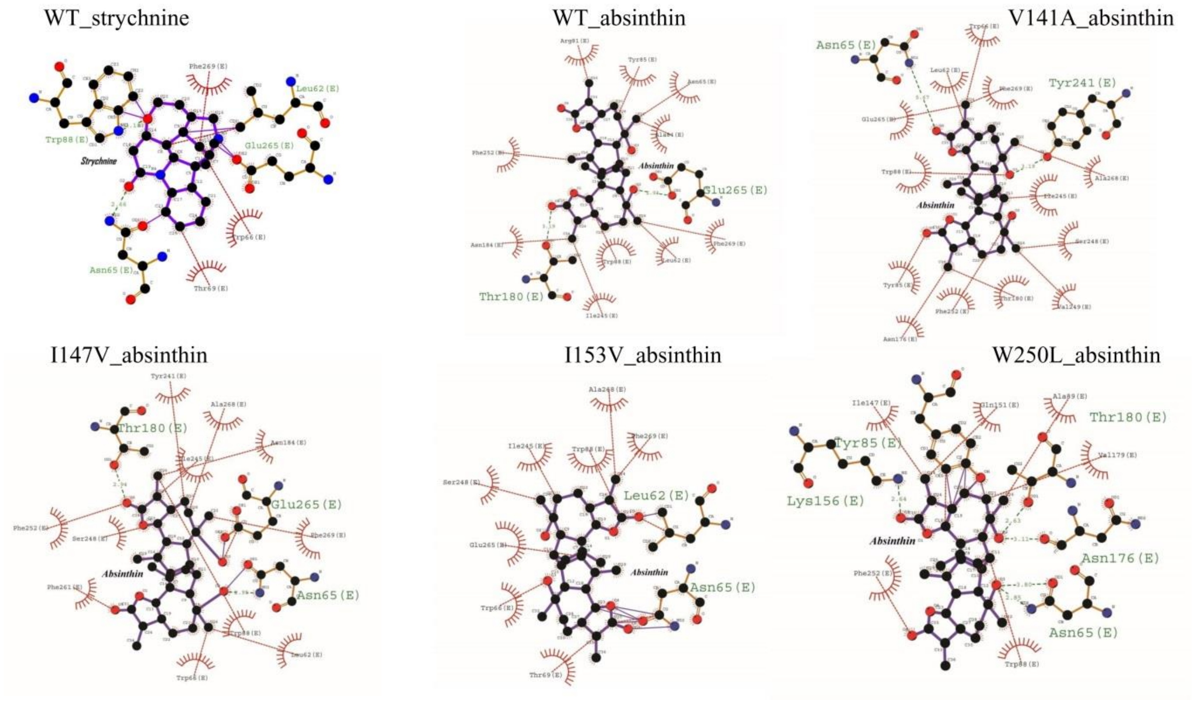

Figure S5. Interaction of modelled TAS2R46 with strychnine and absinthin in orthosteric site. This figure depicts the modes of interactions of the modelled structure of TAS2R46 (wild type and mutants) with strychnine and absinthin in orthosteric site. The ligplot analyses show the hydrogen bonding (green dashed lines) and hydrophobic (red dot) interaction profiles of the wild type and mutant TAS2R46 proteins with the ligand.

Supplementary figure 6

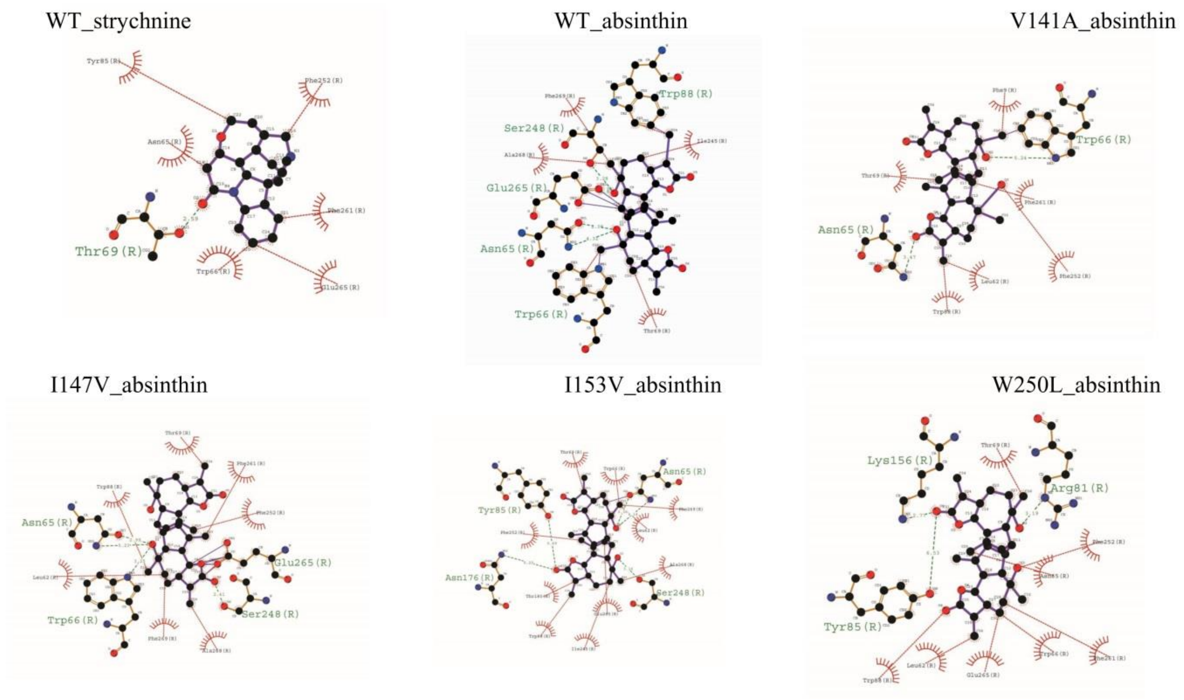

Figure S6. Interaction of 7XP6 TAS2R46 with strychnine and absinthin in vestibular site. This figure depicts the modes of interactions of the cryo EM structure of TAS2R46 (wild type and mutants) with strychnine and absinthin in the vestibular site. The ligplot analyses show the hydrogen bonding (green dashed lines) and hydrophobic (red dot) interaction profiles of the wild type and mutant TAS2R46 proteins with the ligand.

Supplementary figure 7

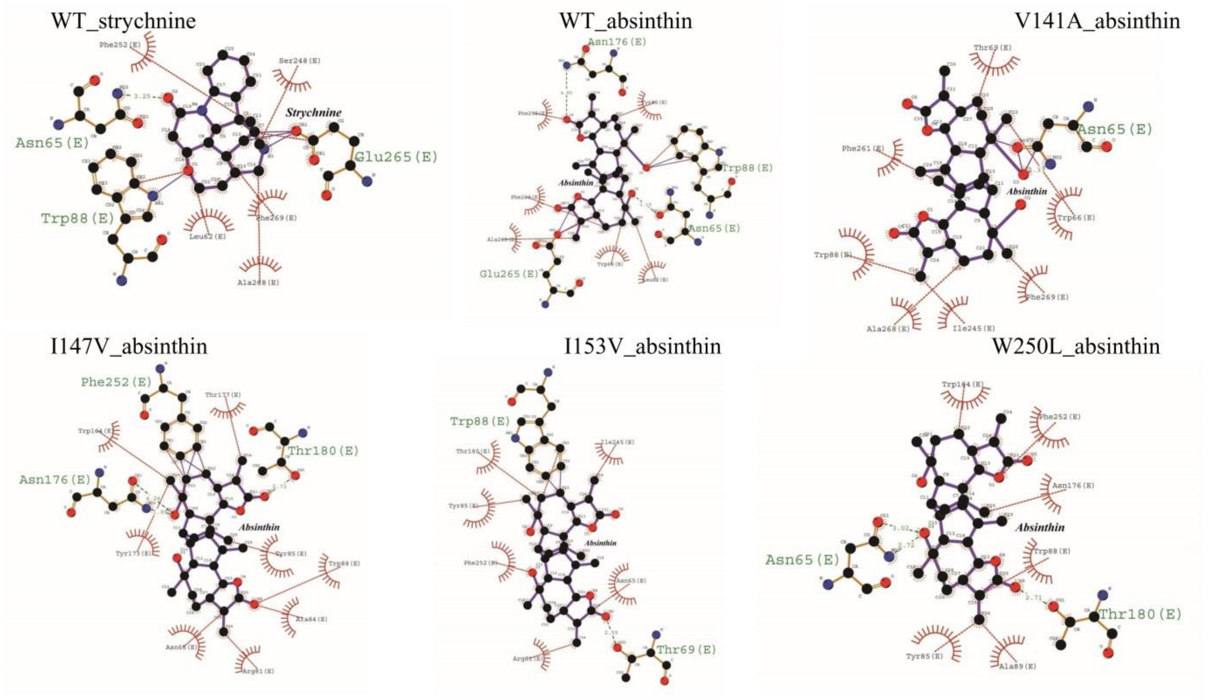

Figure S7. Interaction of modelled TAS2R46 with strychnine and absinthin in vestibular site. This figure depicts the mode of interaction of the modelled structure of TAS2R46 (wild type and mutants) with Strychnine and Absinthin in vestibular site. The ligplot analyses show the hydrogen bonding (green dashed lines) and hydrophobic (red dot) interaction profiles of the wild type and mutant TAS2R46 proteins with the ligand.
